# Therapeutic Effect and Mechanism of Negative Pressure Wound Therapy with Huoxue Shengji Decoction Instillation for Chronic Skin Ulcers

**DOI:** 10.1155/2022/5183809

**Published:** 2022-06-22

**Authors:** Sunfeng Pan, Lie Xiong, Zhenjun Wang, Yujuan Su, Gaofeng Fang, Minda Zhu, Jing Wang, Hanqiang Shi, Shihui Zhu, Yanbo Shi

**Affiliations:** ^1^Department of Burns and Plastic Surgery, Zhejiang Chinese Medical University Affiliated Jiaxing TCM Hospital, Jiaxing, Zhejiang, China; ^2^Jiaxing Key Laboratory of Diabetic Angiopathy Research, Jiaxing, Zhejiang, China; ^3^Jiaxing Burn and Wound Repair Therapy Center, Jiaxing, Zhejiang, China; ^4^Central Laboratory of Molecular Medicine Research Center, Zhejiang Chinese Medical University Affiliated Jiaxing TCM Hospital, Jiaxing, Zhejiang, China; ^5^Department of Burns Surgery, Changhai Hospital, Naval Medical University, Shanghai, China

## Abstract

**Background:**

Negative pressure wound therapy (NPWT) with instillation (NPWTi) is a new treatment for chronic skin ulcers (CSUs), but the choice of perfusate is still investigated. The clinical application of Huoxue Shengji (HXSJ) decoction has been proved to promote the formation of granulation. The formation of fresh granulation, angiogenesis, and proliferation of vascular endothelial cells are closely related. The purpose of this study was to observe the clinical efficacy of NWPT with HXSJ decoction instillation in the treatment of CSUs and to explore the potential mechanism by which HXSJ decoction promotes proliferation of vascular endothelial cells at the cellular level.

**Methods:**

In the clinical study, the random number table was used to divide the patients into three groups (patients were numbered by visit time and assigned a random number and grouped by the remainder after the random number was divided by 3, and when the number of patients in one group reached 20, the enrolment of this group is stopped), including NPWT combined with HXSJ decoction instillation (group *A*), NPWT combined with normal saline instillation (group *B*), and NPWT (group *C*). Related indexes were examined, including the wound cavity volume, bacterial culture, histopathology examination, time periods of debridement, repair methods, and the time of ulcer healing. In the basic research, the effect of HXSJ decoction on the proliferation of HUVECs was analysed by CCK-8 assay and RT-PCR and western blot were used to quantify the VEGF and VEGFR-2 expression in the relevant signalling pathway.

**Results:**

There was no significant difference in the improvement rate of invasive cavity volume (*P* > 0.05) between groups *A* and *B*, but a significant difference was observed between groups *A* and *C* (*P* < 0.05). There was no significant difference in microbial reduction among groups (all *P* > 0.05). Histopathological examination showed that the microvascular count in group *A* was significantly higher than that in groups *B* and *C* (both *P* < 0.01) and there was no statistical difference between groups *B* and *C* (*P* > 0.05). There were no significant differences in the number of invasive lesions and repair methods among the groups (all *P* > 0.05). The healing time of group A was significantly faster than those of groups *B* and *C* (compared to group *B*, *P* < 0.05; compared to group *C*, *P* < 0.01), and there was no statistical difference between groups *B* and *C* (*P* > 0.05). In the cellular experiments, concentration screening was performed and 125 *μ*g/mL HXSJ decoction showed the most significant effect on the proliferation of HUVECs and also enhanced the expression of VEGF and VEGFR-2.

**Conclusion:**

HXSJ decoction can enhance the expression of VEGF and VEGFR-2 and promote the proliferation of HUVECs. Treatment with NWPT with HXSJ decoction instillation can further reduce the wound cavity volume; meanwhile, it can promote blood vessel formation in ulcer wounds, thus accelerating the healing of CSUs.

## 1. Introduction

Chronic skin ulcers (CSUs) are pathological changes such as degeneration and necrosis of local tissues caused by the combined effects of systemic and local factors. CSUs are difficult to heal and treat, and the incidence rate increases every year. In particular, pressure skin ulcers, skin ulcers complicated by diabetes, and venous ulcers of the lower extremities are the most common CSUs [1]. As a common and frequently occurring disease, CSUs seriously threaten human health and severe cases may require amputation, cause cancer, and have fatal outcomes [2, 3]. With the migration of the disease and the occurrence of related complications, patients and their families have to bear the huge economic burden and psychological obstacles associated with CSUs.

The understanding and treatment progress of CSUs in modern medicine is mainly reflected in the wet healing theory, application of growth factors, and preparation of wound beds. Negative pressure wound therapy (NPWT) can improve the microcirculation of the wound, drain wound exudate and inflammatory medium continuously, reduce the cavity volume by mechanical pulling under the condition of negative pressure, and promote angiogenesis and the expression of proliferating cytokines. As a result, NPWT has the potential to accelerate wound healing, shows good efficacy in the treatment of CSUs, and has been widely used in the preparation of wound beds [4]. However, in clinical application, NPWT has problems such as poor drainage and risk of infection [5]. For this reason, negative pressure wound therapy with instillation (NPWTi) has gained increasing attention in recent years, but the optimal solution is still being explored and studied [6].

**Figure 1:**
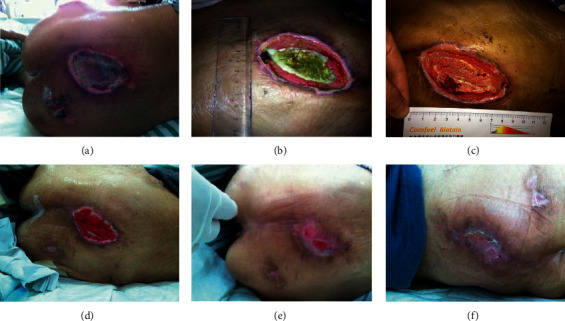
A 92-year-old female patient, with pressure injury in the caudal sacral region involving deep tissue; however, her cardiopulmonary function could not endure the operation. We performed a simple debridement by the bedside followed by a NPWT combined with HXSJ decoction instillation, and the wound healed gradually. (a) Upon admission, the wound depression and purulent exudate accumulated under the skin of necrotic scab, with significant redness and swelling around the wound. (b) After the bedside debridement, the treatment of NPWT combined with HXSJ decoction instillation of activating blood and producing muscle was performed for 15 days, the necrotic tissue on the wound surface was partially dissolved, fresh granulation grew at the edge of the wound, and redness and swelling around the wound subsided. (c) After 30 days of treatment, the necrotic tissue of the wound was basically dissolved and fresh granulation growth could be seen at the base of the wound. (d) After 45 days of treatment, the cavity was completely filled with fresh granulation. (e) After 60 days of treatment, the wound was epithelialized and the wound area was reduced significantly. (f) After 73 days of treatment, the wound healed completely.

Traditional Chinese medicine has a long history of research on CSUs; indeed, some descriptions such as the “Ehythmia,” “Pant mouth sores,” “Skirt wind,” and “Rotten legs” recorded in ancient documents belong to the research field of CSUs. According to the concept of traditional Chinese medicine, CSUs are mainly caused by the collapse of middle-warmer energy (subsidence of the “Ki or breath power”) and obstruction of collaterals, which affect the “Ki” and “blood circulation,” causing blood stasis in the collaterals; the skin loses the nourishment of Ki and blood, followed by the damp invasion of lower energiser, local damage, exposure to insect bites and eczema, and other reasons. The patients are infected with the poison as described above, leading to skin ulcers [7]. Chronic skin ulceratin is associated with poor muscle regeneration; also, the poor muscle regeneration further worsen the chronic skin ulcers. Our previous clinical application of HXSJ decoction on the treatment of chronic skin ulcers improved the formation of fresh granulation, repair of tissue, and promoted wound healing. We have recently used HXSJ decoction instillation combined with NPWT to treat CSUs and achieved significant results (Figure 1). Here, we attempted to explain the therapeutic effect of HXSJ decoction in the treatment of CSUs through the VEGF/VEGFR-2 pathway.

## 2. Materials and Methods

### 2.1. Ethics

The study was conducted in accordance with the tenets of the Declaration of Helsinki and approved by the Ethics Committee of Jiaxing Traditional Chinese Medicine Hospital (REC reference: 2019KY0454).

### 2.2. Clinical Inclusion Criteria

Diagnostic criteria were as follows: (1) the patient entered a pathological inflammatory state caused by various reasons, and the skin ulcer that did not heal and had no healing tendency after treatment for more than 1 month; (2) patients aged 18–75 years; (3) patients with ulcer diameter ≥3 cm and depth ≥0.5 cm; (4) after admission, patients were treated with debridement and NPWTi under anaesthesia.

### 2.3. Clinical Exclusion Criteria

Exclusion criteria were as follows: (1) serious primary diseases of important organs such as the heart, brain, liver, and kidney; (2) pregnant or breastfeeding; (3) abnormal immune or blood coagulation function; (4) mental agitation; (5) treatment was terminated in the event of serious adverse events, sudden deterioration cause by other diseases, or treatments during the period.

### 2.4. General Information and Grouping of Clinical Cases

According to the results of preexperimental and sample size calculation, at least 15 patients in each group are required for this study. Sixty eligible patients from 2019 to 2021 were enrolled in the study (sample size calculation are described in the supplemental materials), including 31 men and 29 women, aged 22–74 years. There were 12, 5, 10, 12, 13, and 8 cases of pressure injury, diabetic foot, lower limb venous ulcers, scar ulcers, traumatic skin ulcers, and other ulcers, respectively. The random number table was used to divide the patients into three groups (patients were numbered by visit time and assigned a random number and grouped by the remainder after the random number was divided by 3, and when the number of patients in one group reached 20, the enrolment of this group was stopped): group *A* was treated with a combination of NPWT with HXSJ decoction instillation; group *B* was treated with NPWT combined with normal saline instillation; and group *C* was treated with NPWT alone.

### 2.5. Preoperative Preparation

All 60 patients with CSUs were evaluated after admission, and a sample for bacterial culture of the ulcerated secretions was collected, followed by disinfection and bandaging. Meanwhile, systemic supportive treatments such as anti-infection therapy, control of blood pressure and blood glucose, nutritional support, and improvement of microcirculation were performed to improve preoperative preparation.

### 2.6. Methods of Wound Debridement

After excluding contraindications, surgical debridement was performed under anaesthesia. The granulation of ulcer oedema, necrotic fascia, and interecological tissue were removed. Bleeding was completely stopped, and the ulcer was repeatedly rinsed with normal saline.

### 2.7. NPWTi

Negative pressure wound therapy dressing (Korean CG Bio Co, Cura PUSF) was applied to the wound, and 10 F ventricle drainage tubes were used as flushing pipes within the dressing in groups *A* and *B*. The dressing was cut and adjusted according to the size of the ulcer and filled in the cavity; the outside was covered with film, the vacuum made the dressing adhere to the wound surface, and sustained continuous drainage at a negative pressure of 90–125 mmHg was performed. The amount and characteristics of the drainage fluid were monitored, and the vacuum sealing drain was monitored for blockages.

### 2.8. Preparation of HXSJ Decoction

*Astragalus*(Cat. 170320), *Salvia* (Cat. 170307), *Safflower* (Cat. 170328), and *Liquorice* (Cat. 170301) were purchased from Anhui Jiayou Chinese Medicine Herb Pieces Co., Ltd.; Angelica (Cat. 170202) and *Rhizome Paridis* (Cat. 170305) were purchased from Zhejiang Chinese Medical University Medical Pieces Co., Ltd.; *Frankincense* (Cat. 170224), *Bletilla* (Cat. 170112), and *Myrrh* (Cat. 170230) were purchased from Jiaxing Oriental Chinese Medicine Decoction Pieces Co., Ltd.; and *Angelica* (Cat. 44170201) was purchased from Hangzhou Mintai Traditional Chinese Medicine Decoction Pipe Co., Ltd.

The components included *Astragalus* (15 g), *Angelica* (20 g), *Salvia* (20 g), *Safflower* (10 g), *Frankincense* (10 g), *Myrrh* (10 g), *Angelica Dahurica* (10 g), *Bletilla striata* (10 g), *Rhizoma paridis* (10 g), and *Liquorice* (6 g). The components were decocted with 1,000 mL of water to prepare 300 mL of decoction. The decoction was taken out, and the components were decocted with additional 800 mL of water to prepare 200 mL of decoction; the decoctions were mixed twice, concentrated to 200 mL filtered with a fine mesh, and then used for instillation of the HXSJ decoction. For experimental studies, the HXSJ decoction was further concentrated to 0.25 g/mL, and the solution was filtered through a 0.22 *μ*m membrane filter and stored at −20°C for later use.

### 2.9. Method of Instillation

In group *A*, 200 ml of HXSJ decoction was dripped into the wound negative pressure drainage dressing to swell, where it was retained for 15 min. After that, the NPWT was continued until the HXSJ decoction was rinsed and NPWTi was performed twice a day. In group *B*, 250 mL of normal saline was used instead of the HXSJ decoction for instillation. Treatment was performed twice daily. The retention time was 15 minutes.

### 2.10. Stage-Two Surgical Repair

After 5 days of treatment with instillation combined with continuous negative pressure drainage, the dressings were removed under anaesthesia. Skin grafts and flaps were used when ulcer granulation was fresh, and no infection was observed. But this was continued in the event of any of the following until the symptoms disappeared: necrotic tissue persisted in the wound cavity, granulation was grey, oedema was observed, the tissue around the ulcer was red and swollen, there was an increase in temperature, and fetid or purulent secretions appeared. Subsequently, skin grafting or skin flap transplantation was completed.

## 3. Observation Indexes

### 3.1. Wound Cavity Volume Measurement

According to the reported and modified method [8], the wound cavity volume was measured by using a syringe drop to fill the wound with saline, until the saline almost reached the edge of the wound; the amount of saline used was then obtained and recorded as the wound cavity volume.

### 3.2. Wound Cavity Recovery Rate

The volume of the wound cavity in each group was measured before and on the 5th day after the first debridement of the ulcer, and the improvement rate of the wound cavity volume was calculated using the method of water injection: cavity recovery (improvement) rate = (preoperative traumatic volume − postoperative traumatic volume on the 5th day)/preoperative traumatic volume × 100%.

### 3.3. Ulcer Bacterial Culture

Ulcer exudate samples were collected for bacterial culture in each group before and on the 1st and 5th days after the first debridement surgery.

### 3.4. Microvessel Counting

The granulation tissue of each group was cut for microvessel counting before and on the 5th day after the first debridement, and the ulcerated granulation tissue was removed for microvessel counting in each group. A single endothelial cell or endothelial cell cluster dyed brown was counted as a vessel. The three areas with the highest microvessel density were selected at low magnification (40x), the number of microvessels in five fields was counted at high magnification (200x), and the average number of microvessels was taken as the number of microvessels in this specimen.

### 3.5. Ulcer Healing

The time of surgical expansion, the method of repair after negative bacterial culture of ulcer granulation, and the healing time of complete epithelialisation of ulcer exudation and absorption were recorded.

## 4. Experimental Study

### 4.1. Reagents and Instruments

The reagents and instruments used in this study were as follows: Dulbecco's modified Eagle's medium (DMEM, Gibco) (10569010, Gibco, USA), foetal bovine serum (10099141C, Gibco, Australia), trypsin-EDTA solution (E607001-0100, BBI, China), penicillin-streptomycin (B540732-0010, Sangon Biotech, China), Cell Counting Kit-8 (CCK-8; E606335, BBI, China), BCA Protein Assay Kit (C503051, Sangon Biotech, China), PrimeScript™ RT reagent kit (RR047A, TaKaRa, Japan), TB Green® Premix Ex Taq™ II (RR820A, TaKaRa, Japan), human VEGF ELISA kit (ml064281, Shanghai mlbio Co., Ltd.), 370-Forma™ Steri-Cycle™ CO_2_ Incubator (Thermo, USA), Inverted Fluorescence microscope (Axio Observer D1, ZEISS, Germany), and Multiskan Spectrum Microplate Spectrophotometer (Multiskan GO, Thermo, USA).

### 4.2. Cell Culture and Grouping

Human umbilical vein endothelial cells (HUVECs) were obtained from the National Experimental Cell Resource Sharing Platform (BMCR, Beijing, China). The cells were cultured in DMEM with a high-glucose-content medium (4.5 g/L, Gibco) supplemented with 10% heat-inactivated foetal calf serum (Gibco) and 1 × penicillin-streptomycin (Sangon Biotech) and incubated at 37°C in a humidified atmosphere containing 5% CO_2_. The experiment was divided into a control group and an HXSJ decoction group, which contained three concentrations (62.5, 125, and 250 *μ*g/mL, respectively). HUVECs were digested with 0.05% trypsin, and trypan blue staining was used to count the cells before adjusting to the appropriate density. The cells (6 × 10^3^ cells/well) were inoculated into a 96-well plate, or 2 × 10^5^ cells/well were inoculated into a 6-well plate. When using the 96-well plate, 100 *μ*L PBS was added to the edge wells to avoid edge effects.

### 4.3. Effect of HXSJ Decoction on HUVEC Proliferation

The HUVECs were seeded into 96-well plates. After adherence, the cells were cultured in a serum-free medium for 24 h. After serum starvation, the HUVECs were changed to DMEM and used for synchronous treatment for 24 h. Finally, the culture medium was replaced with DMEM containing 10% FBS, and different concentrations of HXSH decoction were added. Each group had six replicates. At 0, 24, 48, and 72 h, a CCK-8 assay was performed and the cell proliferation rate was calculated at 100% for the same group at 0 h: cell proliferation rate = *A*_each time point_/A_0 *h*_ × 100%.

### 4.4. Effect of HXSJ Decoction on VEGF Secretion by HUVECs

HUVECs were inoculated in 6-well plates, allowed to culture until adherent, and then changed to serum-free DMEM before adding different concentrations of HXSJ decoction. Each group had six replicates. After treatment for 24 h, the supernatant of the cell culture medium was collected and centrifuged at 12000 × *g* for 15 min. The concentration of VEGF-A in the supernatant was detected using a human VEGF-A ELISA detection kit. At the same time, the cells were digested with 0.05% trypsin and stained with trypan blue. The amount of VEGF-A secreted was calculated and expressed as pg/10^4^ cells.

### 4.5. RNA Isolation and Relative Quantitative RT-PCR

RNA was extracted using the SuperfecTRI Total RNA Isolation Reagent (Sangon Biotech, Shanghai, China). The reaction conditions are presented in Table 1. The primer sequences used in this study are listed in Table 2.

### 4.6. Western Blotting Analysis

Western blotting was performed as previously described with the following primary antibodies: anti-human VEGF-A (1 : 500, ab53465, Abcam, USA), anti-VEGFR-2 (1 : 1000, ab39256, Abcam, USA), goat anti-rabbit IgG H&L secondary antibody (ab6721, Abcam, USA), and anti-*β*-actin (BK7018, Bioker Biotechnology, China). Subsequently, goat anti-mouse IgG or goat anti-rabbit IgG (Pierce Biotechnology, Inc., Rockford, IL, USA) secondary antibodies conjugated to horseradish peroxidase were used. Finally, the blots were detected using an ECL detection system (C510043, Sangon Biotech, China), Mini-PROTEAN® Tetra Cell (Bio-Rad, USA), and Tanon 5200 luminous imaging system (Tannon, China).

## 5. Statistical Analysis

The data were analysed using SPSS 19.0, the Shapiro–Wilk test was used for normality test, and normal distribution data were presented as the mean ± standard error ($\bar{x}\;\pm \;s$). Group differences were analysed by one-way ANOVA, followed by Bonferroni or Tamhane's *T*2 post hoc test according to the variance homogeneity test result. The matched-samples *t* test was used to analyse intragroup comparison before and after treatment. Categorical data were analysed by the chi-square test. Where applicable, groups are labelled with letters to indicate significant differences (different letters denote a significant difference). Statistical significance was defined as *P* < 0.05.

## 6. Results

### 6.1. Safety Assessment

There were no serious adverse events, complications, and sudden deterioration occurred in all patients in this study, and the wounds of all patients healed.

### 6.2. Comparison of the General Data of Clinical Cases

To study the clinical function of the combination of HXSJ decoction and NPWTi on ulcer healing and wound reconstruction, 60 patients were enrolled in this study. All patients were divided into three groups and treated with different methods; there was no statistically significant difference in sex, age, and etiological distribution among the three groups of patients (all *P* > 0.05). See Table3 for further details.

### 6.3. Influence of HXSJ Decoction Combined with NPWT Instillation on the Time of Expanded Wounds, Repair Methods, and Ulcer Healing Time

We found no significant difference in the time of wound reconstruction and repair operation methods among the three groups (all *P* > 0.05). The healing time periods of the three groups were 18.40 ± 3.53 d, 23.20 ± 6.97 d, and 24.25 ± 6.93 d, respectively; group *A* healed significantly faster than groups *B* and *C* (*P* < 0.05; group *B*, *P* < 0.01; group *C*), and there was no statistical difference between groups *B* and *C* (*P* > 0.05), as shown in Table 4.

### 6.4. Effect of HXSJ Decoction Combined with NPWT on Cavity Volume

As shown in Table 5, before the first ulcer debridement, the cavity volume in the three groups was 26.65 ± 1.24 mL (group *A*), 30.65 ± 9.64 mL (group *B*), and 30.40 ± 10.03 Ml (group *C*), respectively. However, 5 days after the first operation, those values were 20.90 ± 9.20 mL, 24.95 ± 8.47 mL, and 25.70 ± 9.11 mL, respectively; the improvement rate of the wound cavity volume was 22.63% ± 9.47%, 19.04% ± 6.40%, and 16.05% ± 6.06%, respectively. The wound cavity volume of all three groups was significantly improved on the 5th day after the first operation (all *P* < 0.05). The improvement rate of the wound cavity volume in group *A* was significantly higher than that in group *C* (*P* < 0.05), but there was no significant difference between groups *A* and *B* (*P* > 0.05).

### 6.5. Effects of HXSJ on Bacterial Infection of Ulcer

Next, we evaluated the effect of HXSJ decoction on the bacterial infection of ulcers. All groups were examined at different time points (the day before the first repair operation of wound reconstruction and the 1st day and 5th day after the operation). As shown in Table 6, the ratios of negative : positive ulcer bacterial infections in group *A* were 5 : 15, 14 : 6, and 18 : 2, respectively; the ratios in group *B* were 7 : 17, 12 : 8, and 16 : 4, respectively; and the ratios in group *C* were 6 : 14, 13 : 7, and 15 : 5, respectively. It showed that ulcer bacterial infections were significantly improved in 5 days after operation (all *P* < 0.05); however, there was no significant difference between the postoperation groups (all *P* > 0.05).

### 6.6. Combined Treatment Promotes the Formation of Microangiogenesis of Ulcer Granulation

Finally, we performed the microvessel counts of ulcer granulation in each group before the first debridement operation and on the 5th day after the operation. As shown in Table 7, the microvessel counts between each group before the operation were 21.50 ± 2.16 (group *A*), 19.95 ± 3.38 (group *B*), and 20.00 ± 3.36 (group *C*)/200 × visual field, respectively, and there was no statistical difference (*P* > 0.05). On the 5th day after the operation, the microvessel counts in each group were 34.50 ± 3.85, 26.25 ± 4.41, and 28.00 ± 5.03/200 × field, respectively. The microvessel count in group *A* was significantly higher than that in groups *B* and *C* (all *P* < 0.01), but there was no statistical difference between groups *B* and *C* (*P* > 0.05). Comparison within the group showed that the microvessel counts were significantly increased at 5 days after surgery compared to preoperative values (all *P* < 0.01). Therefore, microangiogenesis in ulcer granulation was significantly stimulated by HXSJ decoction combined with negative pressure wound therapy.

### 6.7. Effects of HXSJ Decoction on HUVEC Proliferation

**Figure 2:**
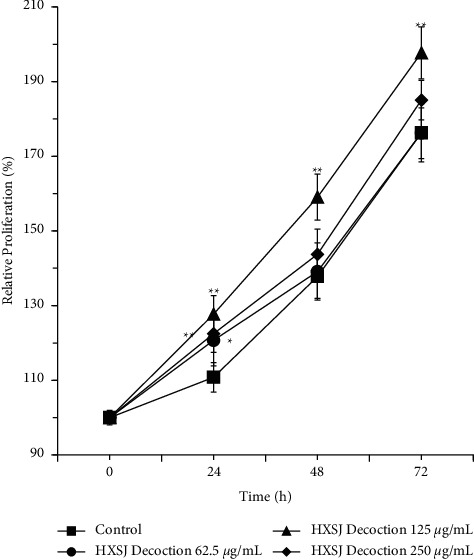
Effects of different concentrations of HXSJ decoction on HUVEC proliferation (^*∗*^*P* < 0.05; ^*∗∗*^*P* < 0.01).

To examine the effect of HXSJ decoction on HUVEC proliferation, we performed the CCK-8 assay. The pre-experiment showed that HUVEC proliferation was significantly promoted in 125 *μ*g/mL HXSJ decoction; therefore, 125 *μ*g/mL was the optimal concentration we selected; meanwhile, 250 *μ*g/mL and 62.5 *μ*g/mL were chosen as double or half. After 24 h of treatment with different concentrations of HXSJ decoction, the cell proliferation rates of groups treated with different concentrations of HXSJ decoction were all significantly higher than that of the control group, as shown in Figure 2; the decoction group (62.5 *μ*g/mL) showed a *P* value <0.05, and the other two groups (125 *μ*g/mL and 250 *μ*g/mL) showed *P* < 0.01. After treatment for 48 and 72 h, the cell proliferation rate after treatment with decoction (125 *μ*g/mL) was significantly higher than that of the control group (all *P* < 0.01); however, no significant difference was observed in any of the other two groups (62.5 *μ*g/mL and 250 *μ*g/mL) when compared to the control group (both *P* > 0.05). This suggests that the HXSJ decoction can promote HUVEC proliferation at an appropriate concentration of 125 *μ*g/mL.

### 6.8. HXSJ Decoction Promotes the Secretion of VEGF-A in HUVECs

**Figure 3:**
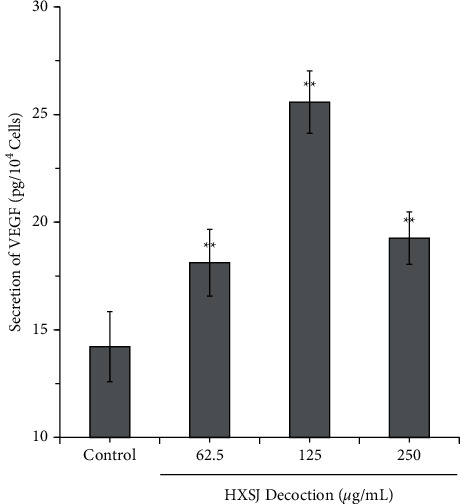
Effects of different concentrations of HXSJ decoction on VEGF secretion in HUVECs (^*∗*^*P* < 0.05; ^*∗∗*^*P* < 0.01).

To examine the effects of the decoction on the secretion of VEGF-A in HUVECs, HUVECs were cultured in a serum-free culture medium, the supernatant was collected, and VEGF-A was detected using a human VEGF-A ELISA kit. Compared with the control group, all three groups treated with different concentrations of HXSJ decoction showed significantly increased VEGF-A secretion levels, reaching 27%, 80%, and 35%, respectively (all *P* < 0.01). In particular, the HXSJ decoction (125 *μ*g/ml) exhibited the most significant effect of stimulating VEGF-A secretion (Figure 3).

### 6.9. HXSJ Decoction Increases VEGF-A and VEGFR-2 Expression Levels in HUVECs at Both mRNA and Protein Levels

**Figure 4:**
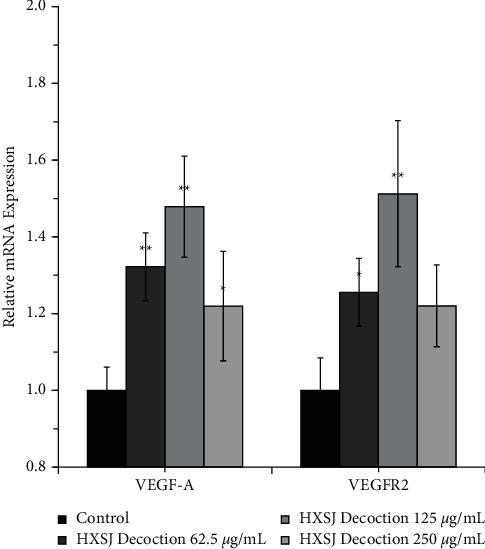
Effects of different concentrations of HXSJ decoction on the expression of VEGF and VEGFR at mRNA levels in HUVECs (^*∗*^*P* < 0.05; ^*∗∗*^*P* < 0.01).

Next, we evaluated the effects of HXSJ decoction on the mRNA and protein expression levels of VEGF-A and VEGFR-2 in HUVECs. After treating HUVECs with different concentrations (62.5, 125, and 250 *μ*g/mL) of HXSJ decoction, the expression of VEGF-A mRNA was upregulated and significantly higher than that in the control group, reaching 32%, 48%, and 22%, respectively. When compared to the control group, the group with 250 *μ*g/mL HXSJ decoction showed *P* < 0.05; however, the other two groups showed *P* < 0.01. It was noticed that HXSJ decoctions (62.5 *μ*g/mL and 125 *μ*g/mL) had significant upregulation effects on the expression of VEGFR-2 mRNA in HUVECs, reaching 26% and 51%, respectively. Compared with the control group, the HXSJ decoction group with 62.5 *μ*g/mL showed *P* < 0.05 and the group with 125 *μ*g/mL showed *P* < 0.01. In comparison, there was no statistical difference between the HXSJ decoction group (250 *μ*g/mL) and the control group (*P* > 0.05), as shown in Figure 4, indicating that HXSJ decoction can upregulate the expression of VEGF-A and VEGFR-2 at mRNA levels in HUVECs, and 125 *μ*g/mL was the most appropriate concentration.

**Figure 5:**
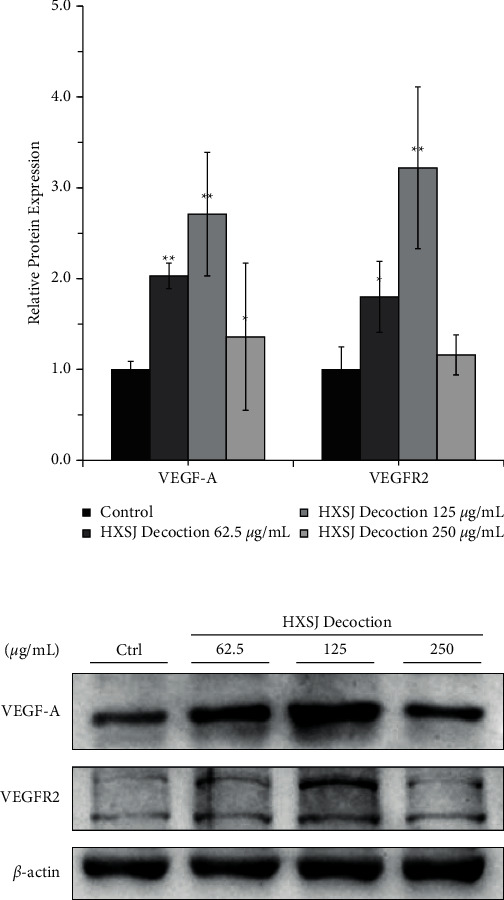
Effects of different concentrations of HXSJ decoction on the expression of VEGF and VEGFR at protein levels in HUVECs (^*∗*^*P* < 0.05; ^*∗∗*^*P* < 0.01).

The western blot results are shown in Figure 5. After treating HUVECs with HXSJ decoction (125 *μ*g/mL), both VEGF-A and VEGFR-2 were significantly increased at the protein level compared to the control group (VEGF-A, *P* < 0.05and VEGFR-2, *P* < 0.01), reaching 271% and 322%, respectively. However, when compared to the control group, all other groups showed no statistical significance (all *P* > 0.05).

## 7. Discussion

**Figure 6:**
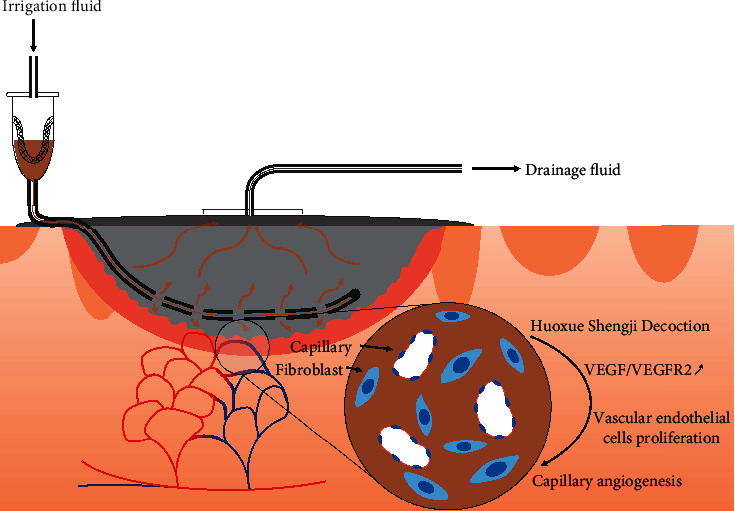
Schematic of NPWT with HXSJ decoction instillation for chronic skin ulcers.

Based on previous studies, we conducted a randomised, controlled trial to further understand the efficacy of HXSJ decoction instillation combined with NPWT in treating CSUs. In the clinical study, as shown in Figure 6, we improved the NPWTi technique, in which the instillation tube was placed in the dressing near the base of the wound cavity so that the decoction could contact the entire wound during the infusion stage. The negative-pressure suction cup was placed in the central area above the dressing, and the liquid continued to flow from the bottom of the dressing to the suction cup during the washing stage to maximise the washing effect of the dressing.

Our results showed that, compared to standard closed drainage treatment, NPWT with instillation could further increase the improvement rate of the wound cavity volume. However, there was no significant difference between the HXSJ decoction and normal saline groups. In our opinion, in addition to the benefits of cyclic cleansing that dilutes and solubilises wound detritus [9], instillation treatment also reduces the accumulation of necrotic tissue in the dressing and helps clear bloody liquid from the drainage tube, thus better maintaining the unobstructed drainage and improving the working efficiency of NPWT [10]. It has been reported that the ulcer infection rate of NPWT is related to bacterial colony accumulation and the use of silver-supplemented NPWTi dressing can effectively reduce the bacterial load [10, 11]; the major components of HXSJ decoction have antibacterial effect. However, there was no difference between the HXSJ and the normal saline groups, which may be caused by the sample size being insufficient.

In our study, we found that when HXSJ decoction instillation was combined with NPWT in the treatment of chronic skin ulcers, ulcer microvascular count was significantly higher than that of treatment with saline instillation combined with standard NPWT. However, the combined treatment did not reduce the number of debridements and simplify the operation procedure but promoted the growth of granulation tissue, thus effectively shortening the time needed for ulcer healing. Many factors affect wound healing, among which angiogenesis plays an important role. The ligand for VEGFR-2 is VEGF-A, and this interaction is important for angiogenesis, which occurs through the coordinate signalling of endothelial-cell proliferation, migration, and recruitment of endothelial cell progenitor cells (EPCs). VEGF-C and VEGF-D bind to VEGFR-3 and are mainly involved in lymphangiogenesis [12]. In our studies, endothelial cells were involved in the blood vessels formation and new blood vessels deliver nutrients and oxygen to the wound site and promote fibroblast proliferation, collagen synthesis, and epithelial regeneration [13]; thus, we chose VEGF-A to evaluate angiogenesis.

HUVECs were used in the cellular experiments to clarify the mechanism of promoting granulation microangiogenesis and accelerating wound healing through HXSJ decoction intervention. The results showed that HXSJ decoction could effectively promote the proliferation of HUVECs and achieved the best effects at a concentration of 125 *μ*g/mL. After 72 h of treatment, the proliferation rate of HUVECs reached 198% ± 7%, which was 13% higher than that of the control group. After 24 h intervention with 125 *μ*g/mL HXSJ decoction, the secretion of VEGF in HUVECs was 26.58 ± 1.45 pg/10^4^ cells, 87% higher than that of the control group. The mRNA expression levels of VEGF-A and VEGFR-2 were increased by 48% and 51%, respectively, and the protein expression levels were increased by 171% and 222% compared to the control group.

Among many factors promoting angiogenesis, VEGF is the most important regulatory factor that induces endothelial cell proliferation [14]. VEGF is expressed by many cells such as endothelial cells, keratinocytes, fibroblasts, fine smooth muscle cells, platelets, central granulocytes, and macrophages [15]. In normal skin, the expression of VEGF is low; when the tissue is injured, VEGF activity increases, and with wound healing, VEGF levels gradually decrease to normal levels [16]. Some studies have shown that VEGF expression in chronic wounds is significantly lower than that in normal skin tissue [17] and wound healing is impeded. Related in vivo experiments have also confirmed that increasing VEGF expression or injection of exogenous VEGF can effectively promote angiogenesis and healing of chronic wounds [15]. Activation of VEGFR-1 can lead to an inflammatory response [18], while VEGFR-2 activation can induce angiogenesis [19]. Meanwhile, some researchers believe that VEGFR-2 plays a more important role in ulcer healing than VEGF and the difficulty of wound healing may be related to the deficiency of VEGFR-2 [20]. Indeed, VEGF and VEGFR-2 are molecular targets for tumour therapy, which inhibit angiogenesis and thus have anticancer effects [21]. Therefore, VEGF and VEGFR-2 can also be used as molecular targets of drugs to promote angiogenesis and accelerate wound healing.

HXSJ decoction is a compound preparation containing a variety of traditional Chinese medicines to promote wound healing. The main components including *Astragalus membranaceus* [22, 23], *Salvia miltiorrhiza* [24], and *Safflower* [25] and their extracts or monomers demonstrated the ability to upregulate the expression of VEGF/VEGFR2 in HUVECs and promote angiogenesis in vitro. The extracts or monomers of *Angelica dahurica* [26], *Frankincense* [27], *Myrrh* [28], and *Bletilla striata* [29] also showed antibacterial and anti-inflammatory effects in promoting wound healing. This is consistent with the theory of traditional Chinese medicine. In the HXSJ decoction, *A. membranaceus* has the effects of supplementing “Ki” and “Yang,” promoting pus discharge and tissue regeneration, used as “king medicine”; *Angelica*, *S. miltiorrhiza*, *Safflower*, *Frankincense*, and *Myrrh* invigorate blood qi, and are also found in the “minister medicine”; *Angelica dahurica* and *Bletilla striata* are “adjuvant drugs” for muscle growth and swelling. *Liquorice* is used to mediate various medicines. The combination can activate blood stasis, induce detumescence, and promote muscle regeneration.

In conclusion, in treating chronic ulcer healing, HXSJ decoction irrigation combined with NPWT effectively promotes angiogenesis and wound healing. In cellular experiments, we focused on exploring the mechanism of HXSJ decoction to accelerate microangiogenesis and wound healing by promoting the expression of VEGF/VEGFR2 in HUVECs.

However, there are several limitations to our work. The sample size included in the study was small, and some data failed to reflect significant differences. In addition to promoting the expression of VEGF/VEGFR2 and accelerating microangiogenesis, HXSJ decoction may also have antibacterial and anti-inflammatory effects; however, these were not discussed in this study and need to be investigated in future work.

## Figures and Tables

**Table 1:** RT-PCR conditions.

Stage	Temperature (°C)	Time (s)	Cycles
Pre-denaturation	95	30	1

PCR	95	5	40
	55	30	
	72	34	

**Table 2:** Sequences of primers for RT-PCR.

Genes	Sequence (5′–3′)	Products size (bp)
GAPDH	F: TGCACCACCAACTGCTTAGC	87
	R: GGCATGGACTGTGGTCATGAG	

VEGF-A	F: GGAGTACCCTGATGAGATCGAG	195
	R: CATTTGTTGTGCTGTAGGAAGC	

VEGFR2	F: GCAGGGGACAGAGGGACTTG	92
	R: GAGGCCATCGCTGCACTCA	

**Table 3:** General information comparison.

Group	Number	Gender	Age	Cause (number)					
		(M/F)	(Yr)	Stress injury	Diabetic foot	Venous leg ulcers	Scar ulcer	Traumatic skin ulcer	Other wounds
*A*	20	11/9	45 ± 15	3	4	3	4	3	3
*B*	20	10/10	45 ± 17	4	0	3	5	7	1
*C*	20	10/10	50 ± 15	5	1	4	3	3	4
*F*/*X*^2^		0.133	0.68	10.612					
*P*		0.935	0.511	0.389					

*Comparison*	*P* value for gender			*P* value for age			*P* value for cause		
*A*-*B*	0.752			1.000			0.232		
*A*-*C*	0.752			1.000			0.742		
*B*-*C*	1.000			0.898			0.397		

Group *A*: HXSJ decoction instillation combined with NPWT. Group *B*: continuous saline instillation combined with NPWT. Group *C*: NPWT. ^*∗*^*P* < 0.05 between groups *A* and *C*. ^*∗∗*^*P* < 0.01 between groups *A* and *C*. Δ*P* < 0.05 between groups *A* and *B*. ΔΔ*P* < 0.01 between groups *A* and *B*. ^#^*P* < 0.05 between groups *B* and *C*. ^##^*P* < 0.05 between groups *B* and *C*.

**Table 4:** Comparison of three different treatments.

Group	Number	Time of wound enlarging	Repair operation method (skin/skin flap)	Healing time (d)
*A*	20	1.30 ± 0.57	17/3	18.40 ± 3.53^*∗∗*^Δ
*B*	20	1.65 ± 0.81	15/5	23.20 ± 6.97Δ
*C*	20	1.75 ± 0.91	16/4	24.25 ± 6.93^*∗∗*^
*F*/*X*^2^		1.845	1.625	5.353
*P*		0.167	0.732	0.007

Comparison	*P* value for time of wound enlarging	*P* value for repair operation method	*P* value for healing time	
*A*-*B*	0.481	0.429	0.031	
*A*-*C*	0.218	0.677	0.070	
*B*-*C*	1.000	0.705	0.952	

Group *A*: HXSJ decoction instillation combined with NPWT. Group *B*: continuous saline instillation combined with NPWT. Group *C*: NPWT. ^*∗*^*P* < 0.05 between groups *A* and *C*. ^*∗∗*^*P* < 0.01 between groups *A* and *C*. Δ*P* < 0.05 between groups *A* and *B*. ΔΔ*P* < 0.01 between groups *A* and *B*. ^#^*P* < 0.05 between groups *B* and *C*. ^##^*P* < 0.05 between groups *B* and *C*.

**Table 5:** Effects of different treatments on the rate of wound cavity volume improvement.

Group	Number	Wound cavity volume				The rate of wound cavity volume improvement (%)
		Before operation (mL)	After operation 5 d later (mL)	*t*	*P*	
*A*	20	26.65 ± 10.24	20.90 ± 9.20	7.814	≤0.001	22.62 ± 9.48^*∗*^
*B*	20	30.65 ± 9.64	24.95 ± 8.47	11.699	≤0.001	19.04 ± 6.40
*C*	20	30.40 ± 10.03	25.70 ± 9.11	8.728	≤0.001	16.05 ± 6.06^*∗*^
*F*		1.009	1.672			3.893
*P*		0.371	0.197			0.026

Comparison	*P* value for wound cavity volume					*P* value for the rate of wound cavity volume improvement
	Before operation			After operation 5 d later		
*A*-*B*	0.630			0.471		0.404
*A*-*C*	0.718			0.284		0.022
*B*-*C*	0.630			1.000		0.629

Group *A*: HXSJ decoction instillation combined with NPWT. Group *B*: continuous saline instillation combined with NPWT. Group *C*: NPWT. ^*∗*^*P* < 0.05 between groups *A* and *C*. ^*∗∗*^*P* < 0.01 between groups *A* and *C*. Δ*P* < 0.05 in groups *A* and *B*. ΔΔ*P* < 0.01 between groups *A* and *B*. ^#^*P* < 0.05 between groups *B* and *C*. ^##^*P* < 0.05 between groups *B* and *C*.

**Table 6:** Effects of different treatments on the bacterial infection of ulcer.

Group	Number	Before the first operation (negative/positive)	The 1st day after the operation (negative/positive)	The 5th day after the operation (negative/positive)	*X * ^2^	*P*
*A*	20	5/15	14/6	18/2	18.754	≤0.001
*B*	20	7/13	12/8	16/4	8.366	0.015
*C*	20	6/14	13/7	15/5	9.095	0.011
*X * ^2^		0.476	0.440	1.558		
*P*		0.788	0.803	0.459		

Comparison	*P* value for bacterial infection of ulcer					
	Before the first repair and reconstruction operation		The 1st day after the operation		The 5th day after the operation	
*A*-*B*	0.490		0.507		0.376	
*A*-*C*	0.723		0.736		0.212	
*B*-*C*	0.736		0.744		0.705	

Comparison	*P* value for bacterial infection of ulcer					
Group	Before-1st day after		Before-5th day after		1st day after-5th day after	
*A*	0.004		≤0.001		0.114	
*B*	0.113		0.004		0.168	
*C*	0.027		0.004		0.490	

Group *A*: HXSJ decoction instillation combined with NPWT. Group *B*: continuous saline instillation combined with NPWT. Group *C*: NPWT. ^*∗*^*P* < 0.05 between groups *A* and *C*. ^*∗∗*^*P* < 0.01 between groups *A* and *C*. Δ*P* < 0.05 between groups *A* and *B*. ΔΔ*P* < 0.01 between groups *A* and *B*. ^#^*P* < 0.05 between groups *B* and *C*. ^##^*P* < 0.05 between groups *B* and *C*.

**Table 7:** Effects of different treatments on microangiogenesis of ulcer granulation.

Group	Number	Microvessel count (pieces/200x field of vision)		*t*	*P*
		Before the first repair and reconstruction operation	The 5th day after the operation		
*A*	20	21.50 ± 2.16	34.50 ± 3.85^*∗∗*^^ΔΔ^	−13.825	≤0.001
*B*	20	19.95 ± 3.38	26.25 ± 4.41^ΔΔ^	−5.570	≤0.001
*C*	20	20.00 ± 3.36	28.00 ± 5.03^*∗∗*^	−6.107	≤0.001
*F*		1.701	17.050		
*P*		0.192	≤0.001		

Comparison	*P* value for microvessel count				
	Before the first repair and reconstruction operation		The 5th day after the operation		
*A*-*B*	0.330		≤0.001		
*A*-*C*	0.365		≤0.001		
*B*-*C*	1.000		0.657		

Group *A*: HXSJ decoction instillation combined with NPWT. Group *B*: continuous saline instillation combined with NPWT. Group *C*: NPWT. ^*∗*^*P* < 0.05 between groups *A* and *C*. ^*∗∗*^*P* < 0.01 between groups *A* and *C*. ^Δ^*P* < 0.05 between groups *A* and *B*. ^ΔΔ^*P* < 0.01 between groups *A* and *B*. ^#^*P* < 0.05 between groups *B* and *C*. ^##^*P* < 0.05 between groups *B* and *C*.

## Data Availability

The data used and/or analysed during the current study are included within this published article and also available from the corresponding author on reasonable request.

## Supplementary Materials

Supplementary Materials Estimation of clinical sample size: two indexes “improvement rate of wound cavity volume” and “microvessel count on the 5th day after the first debridement” of 5 patients in each group were used for the sample size estimation; it turned out that at least 15 patients were needed in each group. Considering other factors such as clinical case dropout, 20 patients were included in each group.

## References

[1] Nicholas M. N., Yeung J. (2017). Current status and future of skin substitutes for chronic wound healing. *Journal of Cutaneous Medicine and Surgery*.

[2] Da Silva L. P., Reis R. L., Correlo V. M., Marques A. P. (2019). Hydrogel-based strategies to advance therapies for chronic skin wounds. *Annual Review of Biomedical Engineering*.

[3] Abdi M. A., Yan M., Hanna T. P. (2020). Systematic review of modern case series of squamous cell cancer arising in a chronic ulcer (marjolin’s ulcer) of the skin. *JCO Global Oncology*.

[4] Sibbald R. G., Mahoney J., V A C Therapy Canadian Consensus Group (2003). A consensus report on the use of vacuum-assisted closure in chronic, difficult-to-heal wounds. *Ostomy/Wound Management*.

[5] Davis K. E., La Fontaine J., Farrar D. (2020). Randomized clinical study to compare negative pressure wound therapy with simultaneous saline irrigation and traditional negative pressure wound therapy for complex foot infections. *Wound Repair and Regeneration*.

[6] Burusapat C., Sringkarawat S. (2021). Efficacy of negative-pressure wound therapy with tetrachlorodecaoxygen-anion complex instillation compared with standard negative-pressure wound therapy for accelerated wound healing: a prospective, randomized, controlled trial. *Plastic and Reconstructive Surgery*.

[7] Li D., Lu J. (2010). *Practical Traditional Chinese Surgery*.

[8] Little C., McDonald J., Jenkins M. G., McCarron P. (2009). An overview of techniques used to measure wound area and volume. *Journal of Wound Care*.

[9] Kim P. J., Attinger C. E., Constantine T. (2020). Negative pressure wound therapy with instillation: international consensus guidelines update. *International Wound Journal*.

[10] Woodmansey E. J., Roberts C. D. (2018). Appropriate use of dressings containing nanocrystalline silver to support antimicrobial stewardship in wounds. *International Wound Journal*.

[11] Hahn H. M., Lee I. J., Woo K. J., Park B. Y. (2019). Silver-impregnated negative-pressure wound therapy for the treatment of lower-extremity open wounds: a prospective randomized clinical study. *Advances in Skin and Wound Care*.

[12] Breen E. C. (2007). VEGF in biological control. *Journal of Cellular Biochemistry*.

[13] Kant V., Gopal A., Kumar D. (2015). Curcumin-induced angiogenesis hastens wound healing in diabetic rats. *Journal of Surgical Research*.

[14] Tammela T., Enholm B., Alitalo K., Paavonen K. (2005). The biology of vascular endothelial growth factors. *Cardiovascular Research*.

[15] Barrientos S., Brem H., Stojadinovic O., Tomic-Canic M. (2014). Clinical application of growth factors and cytokines in wound healing. *Wound Repair and Regeneration*.

[16] Bao P., Kodra A., Tomic-Canic M., Golinko M. S., Ehrlich H. P., Brem H. (2009). The role of vascular endothelial growth factor in wound healing. *Journal of Surgical Research*.

[17] Ho J., Walsh C., Yue D., Dardik A., Cheema U. (2017). Current advancements and strategies in tissue engineering for wound healing: a comprehensive review. *Advances in Wound Care*.

[18] Yoo S. A., Kwok S. K., Kim W. U. (2008). Proinflammatory role of vascular endothelial growth factor in the pathogenesis of rheumatoid arthritis: prospects for therapeutic intervention. *Mediators of Inflammation*.

[19] Ferrara N. (1999). Vascular endothelial growth factor: molecular and biological aspects. *Current Topics in Microbiology and Immunology*.

[20] Zhou K., Ma Y., Brogan M. S. (2015). Chronic and non-healing wounds: The story of vascular endothelial growth factor. *Medical Hypotheses*.

[21] Ferrara N., Kerbel R. S. (2005). Angiogenesis as a therapeutic target. *Nature*.

[22] Zhang L., Yang Y., Wang Y., Gao X. (2011). Astragalus membranaceus extract promotes neovascularisation by VEGF pathway in rat model of ischemic injury. *Die Pharmazie*.

[23] Zhang Y., Hu G., Li S. (2012). Pro-angiogenic activity of astragaloside IV in HUVECs in vitro and zebrafish in vivo. *Molecular Medicine Reports*.

[24] Song M., Chen L., Zhang L. (2020). Cryptotanshinone enhances wound healing in type 2 diabetes with modulatory effects on inflammation, angiogenesis and extracellular matrix remodelling. *Pharmaceutical Biology*.

[25] Zou J., Wang N., Liu M. (2018). Nucleolin mediated pro-angiogenic role of hydroxysafflor yellow A in ischaemic cardiac dysfunction: post-transcriptional regulation of VEGF-A and MMP-9. *Journal of Cellular and Molecular Medicine*.

[26] Zhang X. N., Ma Z. J., Wang Y. (2017). Angelica dahurica ethanolic extract improves impaired wound healing by activating angiogenesis in diabetes. *PloS One*.

[27] Pengzong Z., Yuanmin L., Xiaoming X. (2019). Wound healing potential of the standardized extract of boswellia serrata on experimental diabetic foot ulcer via inhibition of inflammatory, angiogenetic and apoptotic markers. *Planta Medica*.

[28] Sarbaz Z., Yazdanpanahi Z., Hosseinkhani A., Nazari F., Akbarzadeh M. (2019). The effect of sitz bath of hydro-alcoholic extract of myrrh gum on episiotomy wound healing in nulliparous women. *Journal of Family and Reproductive Health*.

[29] Zhao Y., Wang Q., Yan S. (2021). Bletilla striata polysaccharide promotes diabetic wound healing through inhibition of the NLRP3 inflammasome. *Frontiers in Pharmacology*.

