# Supplementary material for: Therapeutic Effect and Mechanism of Negative Pressure Wound Therapy with Huoxue Shengji Decoction Instillation for Chronic Skin Ulcers
Source: Evid Based Complement Alternat Med. 2022 Jun 22;2022:5183809. doi: 10.1155/2022/5183809 (PMC9242787; doi:10.1155/2022/5183809)
Supplement: Supplementary Materials — Estimation of clinical sample size: two indexes “improvement rate of wound cavity volume” and “microvessel count on the 5th day after the first debridement” of 5 patients in each group were used for the sample size estimation; it turned out that at least 15 patients were needed in each group. Considering other factors such as clinical case dropout, 20 patients were included in each group. [file 5183809.f1.docx]

**1. Formula of sample size calculation and sample information**

**1.1 Formula of sample size calculation**

$$n=\varphi^{2}(\frac{\sum S_{i}^{2}}{g})/[\sum\left( \bar{x_{i}}-\bar{x} \right)^{2}/(g-1)]$$

**1.2 Sample information**

“The rate of wound cavity volume improvement (%)” and “Microvessel count of 5th day after the operation (pieces/200× field of vision)” were included in the sample size calculation.

We conducted pre-experiments and collected 15 samples, 5 samples in each group.

| Group | n | The rate of wound cavity volume improvement (%) | |  | Microvessel count of 5th day after the operation (pieces/200× field of vision) | |
| --- | --- | --- | --- | --- | --- | --- |
|  |  | Average | SD |  | Average | SD |
| A | 5 | 26.75 | 6.29 |  | 35.6 | 3.782 |
| B | 5 | 520.39 | 4.986 |  | 29.8 | 4.494 |
| C | 5 | 17.99 | 8.517 |  | 31 | 5.05 |

**2. Sample size calculation**

**2.1 The rate of wound cavity volume improvement**

$\bar{x_{A}}=26.75$ $s_{A}=6.290$

$\bar{x_{B}}=20.39$ $s_{B}=4.986$

$\bar{x_{C}}=17.99$ $s_{A}=8.517$

*g* is the number of groups, *g*=3

Calculated：

$\bar{x}=21.71$

$\sum s_{i}^{2}=136.9636$

$\sum{(\bar{x_{i}}-\bar{x})}^{2}=40.9824$

As α=0.05, β=0.1 ν_1_=*g*-1=2, ν_2_=∞, get φ=2.52, calculated *n*_(1)_=14;

Asα=0.05, β=0.1, ν_1_=*g*-1=2, ν_2_=*g*(n_(1)_-1)=39, get φ=2.62, calculated *n*_(2)_=15;

*n*_(1)_ and *n*_(2)_ are similar, so each group needs 15 samples.

**2.2 Microvessel count of 5th day after the operation**

$\bar{x_{A}}=35.6$ $s_{A}=3.782$

$\bar{x_{B}}=29.8$ $s_{B}=4.494$

$\bar{x_{C}}=31.0$ $s_{A}=5.050$

*g* is the number of groups, *g*=3

Calculated：

$\bar{x}=32.13$

$\sum s_{i}^{2}=60.00206$

$\sum{(\bar{x_{i}}-\bar{x})}^{2}=18.7467$

As α=0.05, β=0.1, ν_1_=*g*-1=2, ν_2_=∞, get φ=2.52, calculated *n*_(1)_=14;

As α=0.05, β=0.1, ν_1_=*g*-1=2, ν_2_=*g*(n_(1)_-1)=39，get φ=2.62, calculated *n*_(2)_=15;

*n*_(1)_ and *n*_(2)_ are similar, so each group needs 15 samples.

| Group | Gender  （M/F） | Age | Course of disease  (d) | Types of ulcer | Wound cavity volume (mL) | | |  | Bacterial infection | | |  | Microangiogenesis of ulcer granulation (pieces/200× field of vision) | |  | Operation | | Healing time (d) |
| --- | --- | --- | --- | --- | --- | --- | --- | --- | --- | --- | --- | --- | --- | --- | --- | --- | --- | --- |
|  |  |  |  |  | Before  operation | 5^th^ day  after | Improvement  (%) |  | Before  operation | 1^st^ day  after | 5^th^ day  after |  | Before  operation | 5^th^ day  after |  | Number of wound enlarging | Repair operation method |  |
| A | M | 49 | 68 | Other wounds | 38 | 27 | 28.94737 |  | + | + | - |  | 20 | 38 |  | 3 | Skin flap | 28 |
| A | F | 39 | 96 | Venous leg ulcers | 20 | 13 | 35 |  | - | - | - |  | 21 | 36 |  | 1 | Skin | 16 |
| A | F | 55 | 132 | Diabetic foot | 21 | 15 | 28.57143 |  | + | - | - |  | 21 | 37 |  | 1 | Skin | 17 |
| A | M | 48 | 45 | Stress injury | 40 | 31 | 22.5 |  | + | + | + |  | 25 | 38 |  | 2 | Skin | 20 |
| A | M | 62 | 191 | Venous leg ulcers | 32 | 26 | 18.75 |  | + | - | - |  | 21 | 29 |  | 1 | Skin | 19 |
| B | M | 69 | 150 | Traumatic skin ulce | 31 | 27 | 12.90323 |  | - | - | - |  | 16 | 32 |  | 2 | Skin flap | 22 |
| B | F | 56 | 71 | Scar ulcer | 28 | 21 | 25 |  | - | - | - |  | 15 | 30 |  | 1 | Skin | 15 |
| B | M | 32 | 48 | Stress injury | 17 | 13 | 23.52941 |  | + | + | + |  | 19 | 22 |  | 1 | Skin | 17 |
| B | M | 38 | 113 | Stress injury | 22 | 17 | 22.72727 |  | - | - | - |  | 23 | 33 |  | 2 | Skin | 24 |
| B | F | 23 | 100 | Traumatic skin ulce | 45 | 37 | 17.77778 |  | + | + | + |  | 18 | 32 |  | 2 | Skin flap | 30 |
| C | F | 36 | 187 | Scar ulcer | 27 | 24 | 11.11111 |  | + | + | - |  | 22 | 37 |  | 1 | Skin | 20 |
| C | F | 31 | 178 | Stress injury | 46 | 41 | 10.86957 |  | - | - | - |  | 17 | 32 |  | 2 | Skin flap | 32 |
| C | F | 61 | 156 | Stress injury | 23 | 19 | 17.3913 |  | + | - | - |  | 22 | 23 |  | 1 | Skin | 21 |
| C | F | 30 | 159 | Other wounds | 44 | 30 | 31.81818 |  | - | - | - |  | 18 | 32 |  | 3 | Skin | 24 |
| C | M | 56 | 30 | Diabetic foot | 16 | 13 | 18.75 |  | + | + | + |  | 28 | 31 |  | 2 | Skin | 17 |
